# Special Issue: Emerging Paradigms in Insulin Resistance

**DOI:** 10.3390/biomedicines10071471

**Published:** 2022-06-22

**Authors:** J. Jason Collier, Susan J. Burke

**Affiliations:** 1 Laboratory of Islet Biology and Inflammation, Pennington Biomedical Research Center, Baton Rouge, LA 70808, USA; 2 Laboratory of Immunogenetics, Pennington Biomedical Research Center, Baton Rouge, LA 70808, USA

This *Biomedicines* Special Issue was designed to attract articles that focused on different facets of biology relating to insulin resistance, defined as reduced cellular and organismal response to the insulin hormone, and its underlying mechanisms. Studies that centered around the relationship of insulin resistance to other conditions relevant to human disease were also welcomed. Collectively, this Special Issue was intended to contain both review articles, as well as studies with novel data, that offered insights into mechanisms, treatments, and perspectives on this prevalent condition. Indeed, insulin resistance impacts most, if not all, human disease conditions. These include, but are not limited to, aging, cancer, diabetes, dementia, and infectious disease [1,2,3,4].

Elevated fasting insulin is one of the best predictors of eventual progression to Type 2 Diabetes (T2D) [5,6]. Cooper and colleagues present the idea that the regulation of basal insulin secretion involves osteocalcin working in conjunction with GLP-1 [7]. This is proposed to be distinct from the paradigm of glucose-stimulated insulin secretion, which arises through increased glucose metabolism, production of metabolites that regulate membrane ion channels, and promote exocytosis. They also suggest that this possible pathway helps to regulate glucagon output from pancreatic alpha cells. This review article puts forth the idea that regulation of both basal and stimulated insulin secretion is important to understanding hyperinsulinemia, metabolic dysregulation, and progression to T2D.

There are several drugs used to treat insulin resistance in humans, with the intent of preventing the metabolic sequalae that ultimately leads to T2D. Some examples include metformin and pioglitazone [8,9], which are both indicated for use to decrease the risk of progression from insulin resistance and prediabetes to overt T2D. These pharmaceuticals act via distinct mechanisms, but each improves overall health when administered to people with insulin resistance and other symptoms of metabolic syndrome. A study in this issue of *Biomedicines* reveals interesting similarities and key differences between adipose tissue depots from mice and humans given pioglitazone, a thiazolidinedione (TZD) drug.

Two different adipose tissue depots, femoral (representing subcantaeous adipose tissue) and abdominal (representing visceral adipose tissue) taken from human clinical trial participants were compared and also examined alongside similar depots isolated from *db*/*db* mice [10]. In both mice and humans, the subcutaneous white adipose tissue depots responded to pioglitzone by increasing the expression of *Ucp1*, often used as a marker of brown adipose tissue or ‘browning’ of white adipose tissue [11]. In addition, the eWAT in mice and abdominal depot from humans each showed downregulation of the gene encoding interleukin-1 beta, a pro-inflammatory cytokine produced by macrophages, in response to pioglitazone. In mice, the *Cd68* gene, expressed in macrophages, was also decreased after pioglitazone intervention [10]. We interpret these data to indicate that at least part of the mechanism underlying the insulin sensitizing effects of pioglitazone involves remodeling of adipose tissue at the gene expression level, leading to reduced inflammation and increased reesterification of lipid into triglyceride storage. These observations are consistent with improved insulin sensitivity despite weight gain, which is a phenotype observed in both mouse models of obesity (e.g., *db*/*db*) and in humans exposed to TZDs [10,12,13,14,15].

Another study using both mice and human subjects to understand insulin resistance was conducted by Naryzhnaya and colleagues [16]. In this study, the authors report that epicardial adipose tissue hypertrophy is associated with elevated fasting insulin. In addition, these data correlate with severity of coronary artery disease. Moreover, lower serum adiponectin was linked with increased adipocyte size. Thus, alterations in fat tissue surrounding the heart, which appears to be connected with alterations in the circulating hormones insulin and adiponectin, may be a critical factor in determining risk for, or severity of, coronary artery disease.

Following up on factors that regulate insulin abundance, Gonzalez-Casimiro et al., review the historical and contemporary literature surrounding the insulin-degrading enzyme (IDE) [17]. They present the conserved nature of IDE, which is present in microorganisms (e.g., viruses) to mammals (including humans). One of the major functions of IDE is to enzymatically cleave insulin into smaller fragments, based on the tertiary structure of the insulin protein as opposed to sequence-specific recognition of precise amino acids. In addition to degradation of insulin, IDE has several other targets, which include pancreatic proteins glucagon, somatostatin, and amylin. The authors go on to describe the controversies that exist in the literature regarding gene deletion of IDE in mice and the in vivo relevance of IDE to circulating insulin in both standard chow feeding as well as high-fat diet conditions.

Both diet and genetics contribute to the development of obesity and insulin resistance [18]. One gene reported to contribute to obesity and have a strong link with body mass index (BMI) is neuronal growth regulator 1 (NEGR1). In addition to high expression in the brain, NEGR1 is also expressed in adipose tissue [19]. Kaare et al., report that NEGR1 deficient male, but not female, mice on HFD display impaired glucose tolerance [20]. The NEGR1 deficient mice also trend towards increased weight gain, while eating significantly less food than wild-type mice. Interestingly, female, but not male, NEGR1 deficient mice have elevated basal levels of glucose after 6 weeks of high-fat feeding. NEGR1 was shown to play a role in regulating circulating lipids, hepatic lipid content (increased in KOs), as well as a reduction in the cross-sectional area of muscle fibers in NEGR1 deficient male, but not female, mice. In a separate study, NEGR1 deficient mice were shown to also have increased lipid storage in liver, which was consistent with their hyperinsulinemia when compared with wild-type mice [21].

Insulin resistance and hyperinsulinemia are often found together, although there is not universal agreement over which arises first [22]. However, it is clear that prolonged, elevated levels of circulating insulin are associated with metabolic disease and cancer [23]. In addition, hyperinsulinemia is associated with increased risk for, and is predictive of, T2D [5]. In a timely review, Cooper et al., outline the links between bone fragility, increased risk of fractures, and hyperinsulinemia [24]. The authors present an interesting argument that alterations in bone mineralization can be driven by changes in circulating insulin, with part of the mechanism being adipocyte sequestration of Vitamin D.

In addition to changes in bone fragility, osteoarthritis (OA), insulin resistance, and T2D are more common in postmenopausal women than in premenopausal women. To address whether a current drug used to treat OA could also be effective against insulin resistance, Chen et al., used raloxifene in glucosamine exposed, ovariectomized rats [25]. Raloxifine is an estrogen receptor modulator, and by virtue of its ability to activate transcriptional targets of the estrogen receptor, may be effective against the inflammation associated with OA and T2D. In this rat model, markers of improved insulin sensitivity were observed after raloxifene intervention, including restoration of skeletal muscle GLUT-4, reduced liver expression of PEPCK, and normalization of serum glucose and insulin concentrations. Thus, estrogen receptor modulators may be highly effective in multiple conditions associated with diseases of aging.

In a separate study using both rodent models and data from human study participants, Solares et al., noted hyperinsulinemia in patients with acute intermittent porphyria (AIP) [26]. AIP is a metabolic disease that results from reduced activity of key hepatic enzyme (porphobilinogen deaminase) in the heme synthesis pathway. This disease has been associated with hyperinsulinemia for decades [27] and the present study noted a significant prevalence of hyperinsulinemia in Spanish patients with a mutation in the gene encoding porphobilinogen deaminase. A proof of principle study indicated that a fusion protein of insulin/ApoAI reduced the expression of *Alas1*, which encodes an ezyme that synthesizes a metabolic precursor associated with the phenotype of AIP. Thus, the mouse model used in this study suggests that liver-specific targeting of insulin may be one strategy to reduce symptoms associated with AIP.

In summary, hyperinsulinemia and insulin resistance are associated with, and often predictive of, metabolic disease in rodent models and humans. In addition, these two highly prevalent conditions worsen the outcomes of many human diseases. This issue provides a variety of perspectives, as well as novel data, that center around insulin resistance, hyperinsulinemia, and distinct conditions impacted by these ever-increasing disorders. Using mice as experimental models with an eye on translational components offers new insights into both mechanisms and possible new therapies on the horizon.
